# Effects of indoor cooking with liquefied petroleum gas versus solid biomass on mosquito and fly density in households

**DOI:** 10.1038/s41598-025-03573-9

**Published:** 2025-06-04

**Authors:** Ian Hennessee, Miles A. Kirby, Xavier Misago, Jackie Mupfasoni, Jiantong Wang, Jean de Dieu Ntivuguruzwa, Florien Ndagijimana, Ghislaine Rosa, Jennifer L. Peel, Lance A. Waller, Joshua P. Rosenthal, Uriel Kitron, Emmanuel Hakizimana, Thomas F. Clasen

**Affiliations:** 1 Gangarosa Department of Environmental Health, Rollins School of Public Health, Emory University, 1518 Clifton Rd, 30322 Atlanta, GA USA; 2 Department of Global Health and Population, Harvard T.H. Chan School of Public Health, Harvard University, Boston, MA USA; 3 Malaria and Other Parasitic Diseases Division, Biomedical Centre, Rwanda Ministry of Health, Kigali, Rwanda; 4 Department of Biostatistics and Bioinformatics, Rollins School of Public Health, Emory University, Atlanta, GA USA; 5 Eagle Research Center, Kigali, Rwanda; 6 Department of Infectious and Tropical Diseases, London School of Hygiene & Tropical Medicine, London, UK; 7 Department of Environmental and Radiological Health Sciences, Colorado State University, Fort Collins, CO USA; 8 Fogarty International Center, National Institutes of Health, Bethesda, MD USA; 9 Department of Environmental Sciences, Emory University, Atlanta, GA USA

**Keywords:** Malaria, Gastrointestinal diseases, Infectious diseases, Risk factors, Epidemiology, Entomology

## Abstract

Cleaner cooking fuels are increasingly promoted to reduce household air pollution-related health effects, but evidence is limited whether changes in cooking fuels could alter vector behavior and human exposure to vector-borne diseases. In the context of a randomized controlled trial in eastern Rwanda, we evaluated differences in mosquito and fly density in 109 intervention houses which received liquefied petroleum gas (LPG) stoves, a continuous fuel supply, and were encouraged to cook indoors compared to 102 control households which continued cooking with biomass fuels, primarily outdoors. *Anopheles* mosquito densities were similar in the intervention group compared to the control group (RR = 0.92, 95%CI: 0.33–2.55), as were culicine densities (RR = 1.17, 95%CI: 0.83–1.63). In contrast, synanthropic fly densities were 69% lower in intervention households (RR = 0.31, 95% CI: 0.22–0.45). In an exploratory analysis of houses that cooked indoors, *Anopheles* densities were higher but not significantly different in intervention houses compared to control houses, whereas culicine and synanthropic fly densities were similar. In settings where outdoor cooking with biomass fuels is common, switching to indoor cooking with cleaner-burning fuels does not significantly increase indoor exposure to *Anopheles* or culicine mosquitoes, while it could significantly reduce exposure to synanthropic flies in kitchen areas.

**Supplementary Information:**

The online version contains supplementary material available at 10.1038/s41598-025-03573-9.

Clean cooking fuels such as liquefied petroleum gas (LPG) are increasingly promoted to reduce household air pollution (HAP), which is responsible for more than 2.3 million deaths per year^1^. However, traditional biomass fuels are often used as insect repellents, and components of fuel combustion such as carbon dioxide and chemical volatiles are known to influence insect behavior^2^. There is therefore some concern that the replacement of traditional biomass fuels with cleaner-burning fuels could affect mosquito or fly behavior, and potentially alter exposure to vector-borne pathogens^3^.

A variety of experimental and observational studies have shown that smoke from biomass combustion can repel *Anopheles* mosquitoes, which transmit malaria,^4–6^ and *Culex* and *Aedes* mosquitoes, which transmit arboviruses such as West Nile and Dengue^7^. However, until recently no studies had directly investigated the effects of different cooking fuels on mosquito behavior.

We previously conducted an experimental hut pilot study in rural Rwanda to compare the effects of cooking with LPG and traditional biomass fuels on mosquito behavior. Cooking with LPG compared to biomass fuels was associated with substantial increases in household entry and host-seeking by lab-reared *Anopheles* gambiae mosquitoes, whereas mortality was reduced^8^. However, this study was limited in scope and was conducted in controlled experimental conditions with lab-reared mosquitoes. Additional entomological and epidemiological studies in real-world conditions are needed.

Changes in fuel use may also impact synanthropic flies which mechanically vector *Shigella*, *E. coli*, *Salmonella*, and other enteric pathogens^9^. Smoke from burning plant parts is traditionally used to repel synanthropic flies,^10^ and woodsmoke has been experimentally shown to deter *Musca domestica*^11^. Woodsmoke can also reduce Tsetse fly density in field conditions^12^. To our knowledge, no studies have explicitly investigated the impact of changes in cooking fuels on synanthropic fly density or behavior.

Although epidemiological evidence is limited, a cluster randomized controlled trial of cleaner-burning biomass stoves in Malawi reported increased malaria incidence among children in houses that received cleaner burning stoves^13^. A recent case-control study in Guatemala also found that individuals from houses which cooked with fuels other than firewood had an increased risk of arbovirus infection,^14^ although in both cases these were secondary outcomes of health impact evaluations. Epidemiological evidence is limited about the effect of cooking fuels on enteric diseases which can be mechanically vectored by flies.

As cleaner cooking is increasingly promoted to reduce health effects of household air pollution, rigorous studies are needed to characterize potential effects of clean fuel adoption on mosquito and flies and the diseases they transmit^8^. We therefore conducted a study to examine whether the provision of an intervention consisting of LPG stoves and fuel could impact mosquito and fly density among households using biomass at enrollment. A secondary objective was to determine whether the intervention affected reported malaria and diarrhea longitudinal prevalence among household residents.

## Materials and methods

### Study design and setting

We conducted a randomized controlled trial nested within the Rwanda center of the multi-country Household Air Pollution Intervention Network (HAPIN) study, a randomized controlled trial to assess the health effects of an LPG stove and fuel intervention among populations which traditionally rely on solid biomass fuels for cooking^15^ .The HAPIN trial reported high fidelity and adherence of the intervention,^16^ and achieved substantial reductions in PM_2.5_, black carbon and carbon monoxide^17,18^. The trial is registered with clinicaltrials.gov (NCT02944682, 26/10/2016).

The trial took place in Kayonza District, Eastern Province, Rwanda. The majority of the population lives in rural settings, and malaria prevalence was 18% among children 6–59 months in 2017^19^. *An. gambiae* s.l. is the dominant *Anopheles* species. Culicine mosquitoes are also common, particularly *Culex quinquefasciatus*^20.^ The district received indoor residual spraying (IRS) with a neonicotinoid-pyrethroid insecticide combination in April 2019.

## Participants, randomization, and masking

For the current study we included a random sample of houses participating in the HAPIN trial in Rwanda and maintained the randomized trial design of the larger study. The HAPIN trial inclusion criteria are reported elsewhere^15^. Briefly, women between 18 and 35 years old with viable singleton pregnancies between 9 and 20wk gestation who cooked primarily with biomass stoves were considered eligible and enrolled at antenatal care clinics. After baseline surveys were completed, households were randomly assigned by study investigators in a 1:1 ratio to intervention and control arms^21^. Intervention households were provided an LPG stove, a continuous LPG fuel supply for 18 months, and were encouraged to use LPG exclusively for cooking. Intervention participants were also encouraged to cook in a covered location to protect LPG stoves. Control households received no intervention but received the same stove and fuel supply or an alternative of similar value at the end of the trial. Neither participants nor assessors were blinded to the intervention.

## Sample size and eligibility

The sample size for the current study was calculated in order to observe a 25% change in *Anopheles* spp. density, based on previously published estimates of *Anopheles gambiae* s.l. bites/person/night from Kayonza District^20^. With 80% power, α = 0.05, and a correlation coefficient among repeated measurements of 0.6, we estimated a required sample size of 100 households per arm and sought to enroll 110 from each arm in order to account for 10% refusal or loss to follow-up.

Houses that were participating in the HAPIN trial and had been randomized after August 1, 2018 were eligible to be selected such that houses would have at least five remaining months of follow-up by the first round of vector sampling. Houses that had completed the trial, voluntarily exited the trial, or had been enrolled prior to August 1, 2018 were ineligible. 110 intervention and 110 control houses were randomly selected from eligible households using a random number sequence generated by study investigators. Selected houses were visited by a study team who administered information and consent forms to the primary HAPIN participants. Houses were enrolled in the study after participants provided written informed consent.

Because burning biomass indoors may be more likely to repel indoor-biting mosquitoes than burning biomass outdoors or in a separate outdoor kitchen,^8,22^ we hypothesized that the effect of the intervention may be modified by cooking location. Preliminary HAPIN baseline data suggested that cooking inside the main house was uncommon in the study area, so we purposefully selected 40 additional households (20 intervention and 20 control) that reported cooking indoors during HAPIN baseline visits. These houses were enrolled as described above. We conducted and reported all primary analyses with the randomly selected group (group 1), but included this purposefully selected group (group 2) in exploratory analyses of effect modification between cooking location and the intervention.

## Procedures

### Household visits

Three rounds of entomological sampling were conducted: Rounds 1 and 2 were conducted during the short rains in October-December 2019, and Round 3 was conducted during the long rains in March 2020. Study teams visited each house over two consecutive days during each round. Visits were conducted in the morning between 07:00 and 11:00. On the first day study teams administered a brief survey and placed insect traps. They returned to each house the following morning to collect the traps and complete the survey.

### Baseline and visit characteristics

Baseline characteristics of study participants were collected upon enrollment in the HAPIN trial and included maternal age at baseline, gestational age at baseline, and education. Baseline cooking practices were also assessed and included the primary type of fuel used and the location of the primary cooking stove. Baseline housing characteristics included wall, floor, and roofing materials. Baseline environmental characteristics for each household were extracted from geospatial datasets and included household elevation, population density, and proximity to rice fields (Supplementary methods).

Questionnaires were administered to HAPIN participants at each entomological sampling visit. Questionnaires included information about types of fuel used for cooking in the last 24 h, the primary cooking location, and the number of people that slept in the house the night before. Study staff visually assessed the presence of > 1 cm-wide cracks or openings in exterior windows, doors, or walls through which mosquitoes or flies could enter, and checked for the presence of water-holding containers. They visually assessed the presence of toilets or latrines and rubbish piles and measured the distance from these to the primary cooking location. Staff visually assessed the presence of domestic animals in household compounds and animal or human feces around compounds. Participants were asked about LLIN use by household members the night before the survey, whether the household received IRS in the last 12 months, and whether participants had used insecticides or burned any materials to repel mosquitoes or flies in the prior 24 h. Monthly temperature and rainfall were estimated using satellite datasets (Supplementary methods).

## Entomological sampling methods

*CDC light traps*: Miniature CDC light traps (Model 512; John W. Hock Company, Gainesville, FL) were placed at approximately 1.5 m above the foot of the respondent’s bed to collect host-seeking *Anopheles* and culicine mosquitoes. Participants were asked to plug in the battery at 18:00, and leave it running throughout the night until the return of the study team the following morning. Mosquitoes were transported in coolers to a field entomology laboratory.

### Prokopacks

Battery-powered Prokopack vacuum aspirators were used to sample resting mosquitoes in bedrooms, kitchens, and outdoors around the perimeter of each house. Collections from each location were kept in separate containers and transported to the field laboratory.

### Fly traps

Extra-large blue sticky fly traps (Product code 10303, Suterra Ltd, UK) were used to sample domestic synanthropic flies and mosquitoes in cooking areas during each round^23^. Each card was cut in half, and each half was placed at a 45-degree angle 1–2 m from the primary stove location. Traps were retrieved on the following day and transported to the field laboratory.

## Entomological sample processing and identification

All mosquitoes were counted and morphologically identified at the field entomology laboratory using standard keys. All *Anopheles* mosquitoes and blood-fed *Cx. quinquefasciatus* mosquitoes were transported to the Rwanda Biomedical Centre entomology laboratory in Kigali for further processing. All *An. gambiae* s.l. were identified to the species level with polymerase chain reaction (PCR), and all *Anopheles* were tested for *Plasmodium falciparum* presence using enzyme-linked immunosorbent assay (ELISA)^20^. ELISA was also used for bloodmeal analysis of blood-fed mosquitoes.

### HAP sampling

Particulate matter 2.5 (PM_2.5_) concentrations were measured in a subset of 144 houses using Particulate and Temperature Sensors (PATS+, Berkely Air) placed adjacent to CDC light traps. Devices were clean air zeroed according to manufacturing instructions and set to provide PM_2.5_ concentrations (µg/m^3^), temperature (°C), and percent relative humidity (RH) readings every minute from 4pm on the day the CDC light traps were installed until 10am the next morning. This period was chosen to detect HAP peaks associated with fuel usage for evening and morning meal preparation. Values below the limit of detection of 10 µg/m^3^ were recorded as 10 µg/m^3^.

### Outcomes

### Entomological outcomes

We analyzed three primary entomological outcomes. The first outcome, *Anopheles* density, was defined as the total number of *Anopheles* spp. mosquitoes collected via CDC light traps, Prokopacks, and fly traps per house per sampling round. The second outcome, culicine density, was the total number of culicine mosquitoes sampled in CDC light traps. Prokopacks, and fly traps per house per sampling round. Culicine mosquitoes included *Culex* spp., *Aedes* spp., and *Mansonia* spp. mosquitoes. The third outcome, synanthropic fly density, was measured as the number of synanthropic flies collected in primary cooking areas per household per sampling round. Synanthropic flies included all flies in the Muscidae, Calliphoridae, Fanniidae, and Sarcophagidae families, which include the fly species most frequently implicated as mechanical vectors of enteric pathogens.

We also assessed *P. falciparum* infection among *Anopheles* mosquitoes and blood-meal composition of blood-fed *Anopheles* spp. and *Culex quinquefasciatus* mosquitoes as secondary entomological outcomes.

### Secondary epidemiological outcomes

Secondary outcomes included self-reported malaria and diarrhea prevalence among participating mothers and their infants. Malaria and diarrhea outcomes were assessed using data collected as part of the HAPIN trial. After a baseline assessment and randomization into the HAPIN trial, assessments were conducted for pregnant women between 24 and 28 weeks gestation (visit P1) and again between 32 and 36 weeks gestations (visit P2). Three assessments were then conducted after birth when children were approximately 3, 6, 9, and 12 months old (visits B1, B2, B3, and B4). At each visit except for B3, mothers were asked whether they were tested for malaria in the period since the prior visit. We defined longitudinal prevalence of malaria in mothers as the number of periods in which mothers reported having one or more positive malaria tests divided by the number of periods of observation.

At visits B1 through B4, mothers were also asked to report any diagnostically confirmed cases of malaria in their infants since the prior visit, or since birth for the B1 visit. Longitudinal prevalence of malaria in children was defined as above. We also conducted a sensitivity analysis to include only positive malaria tests which study staff were able to verify via clinic cards.

During visits B1 to B4 mothers were asked to report if their infants had diarrhea at any point the past seven days, defined as passage of three or more loose stools within a 24-hour period. Longitudinal prevalence of diarrhea in children was defined as the number of weeks in which infants had one or more reported episodes of diarrhea divided by the number of weeks of observation.

### Statistical analysis

All statistical analyses were conducted with R version 4.0.2 (R Core Team, Vienna Austria) and SAS version 9.4 (SAS Institute, Cary NC). We fit generalized linear mixed effect models to assess the impact of the intervention on densities (rates) of *Anopheles* and culicine mosquitoes and synanthropic flies, with the control arm as the reference group. Vector counts were over-dispersed and were therefore modeled using a negative binomial distribution, and we included random effects to account for repeated observations at the household level^24^. We fit log-binomial models to assess the effect of the intervention on longitudinal prevalence of reported malaria and diarrhea in mothers and children^25^.

We first fit unadjusted models to measure the independent effects of the HAPIN intervention. We then fit multivariate models adjusting for covariates that were imbalanced between study arms and potential confounders. For *Anopheles* and culicine mosquitoes and reported malaria these included maternal education, presence of cracks or openings in houses, insecticide treated net usage, proximity to rice fields, and population density. Potential confounders assessed for flies and reported diarrhea included maternal education, presence of openings in houses, and population density. We assessed potential effect modification of the association between the intervention and each outcome based on the primary cooking location (indoor vs. outdoor/ in a separate cooking structure) on the day that vector sampling was conducted.

### Ethics

This study was reviewed and approved by the Emory Institutional Review Board (IRB 00110407) and the Rwanda National Ethics Committee (IRB 00001497, No.194/RNEC/2019). All study procedures were conducted following the relevant guidelines and regulations.

### Role of the funding source

The funder of the study had no role in study design, data collection, data analysis, data interpretation, or writing of the report.

## Results

### Study population characteristics

**Fig. 1:**
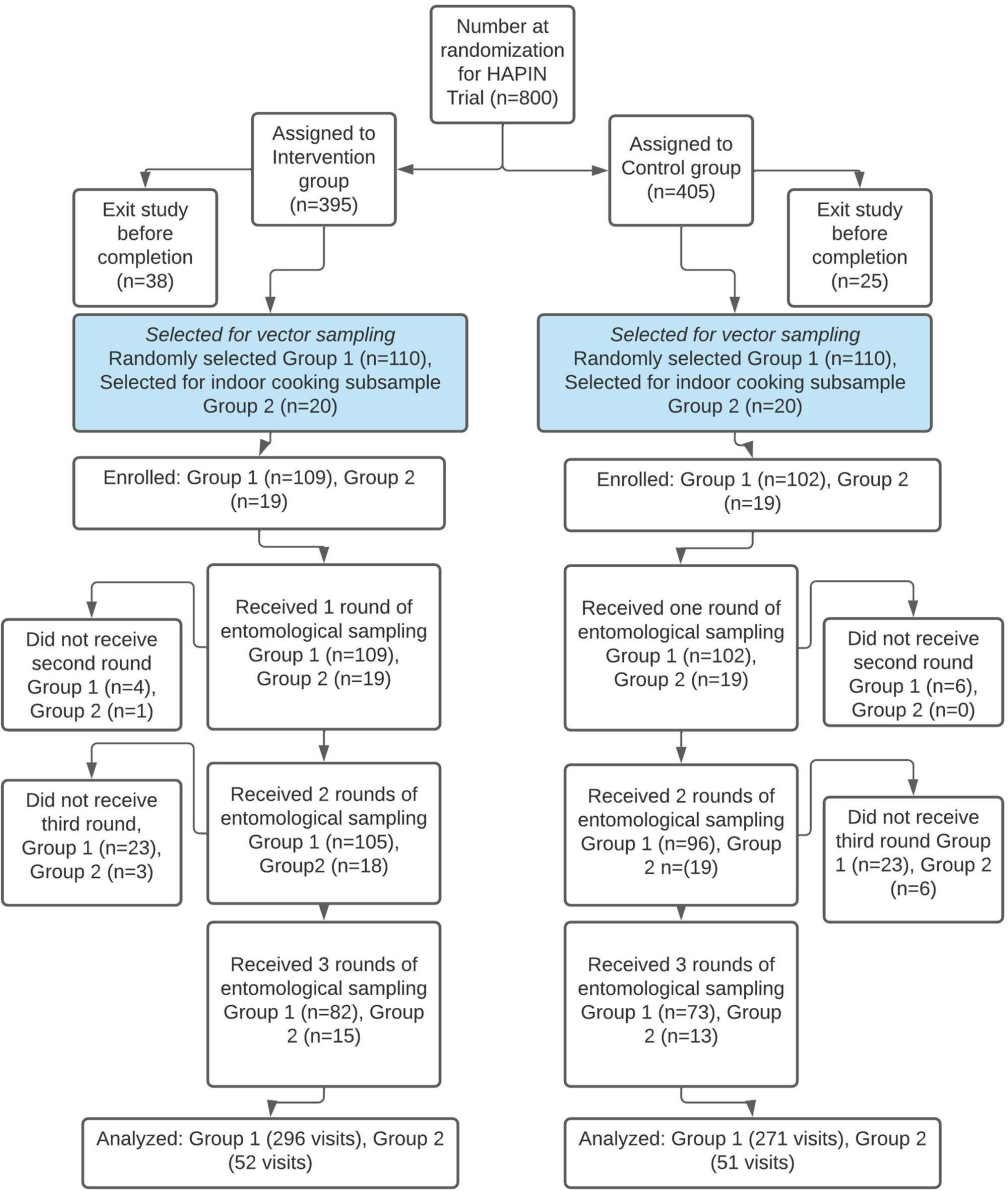
Flow diagram of household selection from HAPIN trial population.

Of 220 group 1 houses that were randomly selected, 102 control and 109 intervention households were enrolled. An additional 38 (19 intervention and 19 control) group 2 households that were purposefully selected based on their cooking location at baseline were also enrolled. Inability to contact households to schedule sampling visits was the primary reason for non-enrollment. All enrolled houses received at least one round of entomological sampling. Five intervention and six control houses did not receive a second round of sampling because they completed and exited the HAPIN trial before the second visit. Sampling was interrupted during the third round due to the COVID-19 pandemic and 26 intervention houses and 29 control houses did not receive a third visit. A total of 567 sampling visits were conducted among group 1 houses, and 103 visits were conducted among group 2 houses (Fig. 1).

Fidelity was high; intervention houses received LPG stoves and fuels a median of 9.5 days after randomization (IQR = 6–16 days), and all houses received the intervention prior to the start of entomological sampling. Maternal age and gestational age at baseline were similar between participants in each arm, whereas fewer mothers in the control arm had completed secondary or further education (Table 1). Wood was the most common fuel source among control and intervention groups at baseline, followed by charcoal. Most participants in both groups reported cooking outdoors or in a separate cooking structure at baseline. Open/3-stone fires were the most common primary stove type in both arms, followed by simple wood-burning stoves called ronderezas and portable charcoal-burning stoves called imbaburas; fewer households in the control arm reported using imbaburas compared to the intervention arm.

**Table 1:** Baseline characteristics of study participants and households.

	Control (*n* = 102)	Intervention (*n* = 109)
Characteristics of women participants		
Maternal age at baseline, mean (SD)	27.3 (4.3)	27.4 (4.2)
Gestational age at baseline, mean (SD)	15.4 (2.9)	15.5 (2.9)
Maternal education, n (%)		
No formal education	5 (4.9)	9 (8.3)
Primary	68 (66.7)	52 (47.7)
Secondary or college	29 (28.4)	48 (44.0)
Number of children under 12 years, mean (SD)	1.52 (0.8)	1.55 (1.0)
Cooking practices at baseline		
Primary fuel used, n (%)		
Wood	86 (84.3)	71 (65.1)
Charcoal	15 (14.7)	37 (33.9)
Other	1 (1.0)	1 (0.9)
Location of primary cooking stove, n (%)		
Outdoors or separate cooking structure	97 (95.1)	99 (90.8)
Indoors in main house	5 (4.9)	10 (9.2)
Type of primary stove, n (%)		
Open/3-stone fire	29 (46.8)	22 (31.9)
Portable charcoal-buring stove (Imbabura)	9 (14.5)	21 (30.4)
Simple wood stove (Rondereza)	19 (30.6)	18 (26.1)
Portable wood stove	5 (8.1)	6 (8.7)
Other	0 (0.0)	2 (2.9)
Characteristics of households		
Wall materials, n (%)		
Mud	53 (52.0)	56 (51.4)
Concrete	15 (14.7)	35 (32.1)
Floor materials, n (%)		
Mud	79 (77.5)	62 (56.9)
Concrete	26 (25.5)	47 (43.1)
Roof materials, n (%)		
Corrugated metal	102 (100.0)	109 (100.0)
Environmental characteristics		
Elevation (m), mean (SD)	1549.6 (100.1)	1584.6 (97.1)
Rice fields within 1 km, n (%)	2 (2.0)	7 (6.4)
Population density / km^2^, mean (SD)	583.6 (385.4)	815.4 (593.7)
Follow-up periods:		
Mothers, mean (SD)	4.6 (0.6)	4.6 (0.6)
Children, mean (SD)	3.6 (0.8)	3.6 (0.7)

Intervention adherence was high; 97% of intervention houses reported using LPG as their primary cooking fuel at follow-up (the day of each visit) and > 99% of control houses reported using biomass as their primary cooking fuel at follow-up. Nearly 90% of control houses reported cooking outdoors or in a separate cooking structure at follow-up, whereas 91% of intervention houses reported cooking inside the main house (Table 2). Mean PM_2.5_ concentrations were 30.7 µg/m^3^ (SD = 26.1) in control bedrooms, which was slightly higher than 25.3 µg/m^3^ (SD = 30.2) measured in intervention bedrooms.

**Table 2:** Characteristics of study participants and households during entomological sampling visits.

	Control (visits = 271)	Intervention (visits = 296)
Cooking practices the night before sampling visit		
Fuel used in primary stove, n (%)		
Biomass	269 (99.6)	9 (3.1)
LPG	1 (0.4)	283 (96.9)
Cooking location for primary stove, n (%)		
Outdoors or separate cooking structure	239 (88.8)	27 (9.3)
Indoors in main house	30 (11.2)	264 (90.7)
Housing characteristics		
People slept in house night before, mean (SD)	3.96 (1.3)	4.02 (1.4)
Cracks or openings in windows, doors, and/or walls, n (%)	199 (73.4)	199 (67.2)
Open, water-holding containers, n (%)	97 (35.8)	102 (34.5)
Toilet/ latrine is covered, n (%)	16 (9.6)	25 (17.0)
Distance (m) from latrine to kitchen, mean (SD)	10.38 (8.5)	13.48 (7.5)
Distance (m) from rubbish pile to kitchen, mean (SD)	8.77 (5.6)	11.61 (9.4)
Domestic animals in compound, n (%)	46 (59.0)	47 (55.3)
Animal/human feces in compound, n (%)	33 (42.3)	26 (30.6)
Vector control activities		
% of occupants that slept under net night before, mean (SD)	65.0 (41.0)	78.0 (35.0)
Received IRS in last 12 months, n (%)	229 (86.4)	252 (87.2)
Used insecticides or burned materials to repel mosquitoes or flies in last 24 h, n (%)	6 (2.2)	11 (3.7)
Indoor conditions		
PM_2.5_ (µg/m^3^) in bedrooms, mean (SD)	30.7 (26.2)	25.3 (30.2)
Temperature (°C) in bedrooms, mean (SD)	23.5 (1.0)	23.4 (1.2)
Relative humidity (%) in bedrooms, mean (SD)	72.8 (3.8)	73.0 (3.2)
Environmental characteristics:		
LST (°C), current month, mean (SD)	27.06 (1.0)	26.94 (1.0)
LST (°C), one-month lag, mean (SD)	30.39 (2.3)	30.13 (2.2)
Rainfall (mm), current month, mean (SD)	130.7 (21.2)	130.4 (20.4)
Rainfall (mm), one-month lag, mean (SD)	101.2 (30.6)	101.8 (30.2)

### Entomological outcomes

#### *Anopheles* mosquitoes

**Fig. 2:**
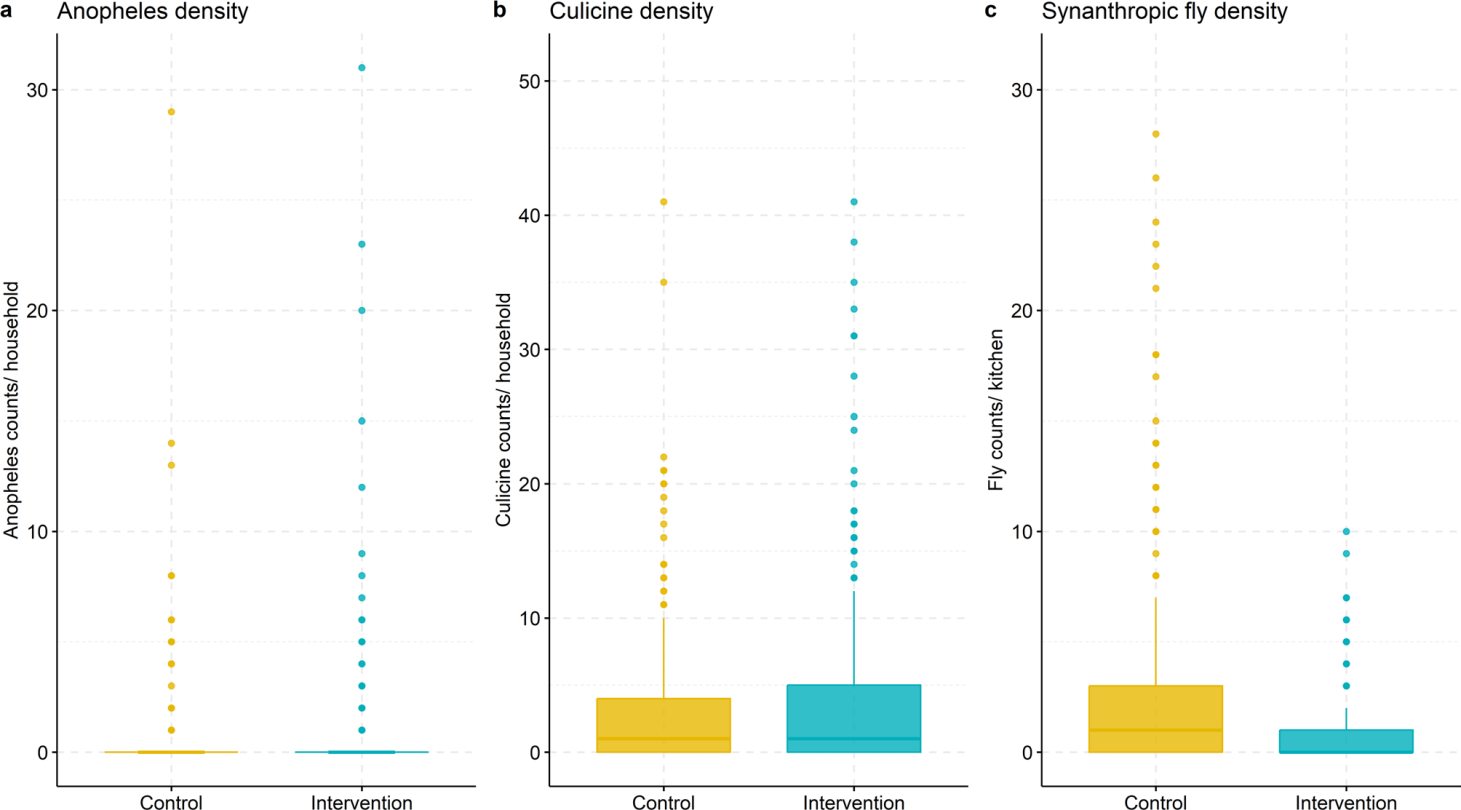
Densities of *Anopheles * mosquitoes (**a**), culicine mosquitoes (**b**) and synanthropic flies (**c**) by intervention status. Medians are shown with solid horizontal lines at the center of each box plot.

We collected 356 *Anopheles* mosquitoes during 567 sampling nights; 336 (94%) were collected via CDC light traps in participant bedrooms and an additional 20 (6%) were collected by Prokopacks, mostly from kitchens (Supplementary Table 1). *An. gambiae* s.l. accounted for 82% of all *Anopheles* collected (Supplementary Table 2). Mean *Anopheles* density was 0.5 (SD = 2.4, median = 0, range 0–31) per sampling night in control houses and 0.7 (SD = 3.0, median = 0, range 0–31) in intervention houses (Table 3; Fig. 2). In both unadjusted and adjusted models, *Anopheles* densities were similar in the intervention compared to the control group (unadjusted rate ratio (RR) = 0.92, 95% CI: 0.33–2.55; adjusted RR = 1.23, 95% CI: 0.51–2.99). Covariate effect estimates are available in Supplementary Table 3.

We then assessed potential effect modification by cooking location, including the group 2 houses. Among houses that cooked indoors, *Anopheles* densities were higher among intervention houses compared to control houses, although confidence intervals were wide and included the null (unadjusted RR = 1.86, 95% CI: 0.18–19.28; adjusted RR = 3.66, 95% CI: 0.92–14.59) (Supplementary Table 4). In contrast, *Anopheles* densities were similar in intervention and control houses that cooked outdoors (unadjusted RR = 0.73, 95% CI: 0.10–5.47; adjusted RR = 1.09, 95% CI: 0.31–3.83). We observed similar effects when we restricted the analysis to just the randomly selected group 1 houses, although the effect estimates were less precise (data not shown). Mean PM_2.5_ concentrations were the highest in control houses which cooked indoors at follow-up (Supplementary Table 5). We observed a negative but not statistically significant association between each standard deviation increase in PM_2.5_ and *Anopheles* densities (RR = 0.65, 95% CI: 0.15–2.82) (Supplementary Table 6).

A single *Anopheles* mosquito from a control household was *P. falciparum* sporozoite-positive, whereas no *Anopheles* from intervention households were positive. Blood-fed status and bloodmeal composition of blood-fed mosquitoes were similar in both groups (Supplementary Table 7).

### Culicine mosquitoes

We collected 2,145 culicine mosquitoes, 2,048 (96%) of which were *Cx. quinquefasciatus* (Supplementary Table 2). Among all culicines, 1920 (90%) were collected via CDC Light traps, 164 (8%) were collected by Prokopacks, and 61 (3%) were collected by sticky fly traps (Supplementary Table 1). Mean culicine density was 3.3 (SD = 5.4, median = 0, range 0–41) per sampling night in control houses, compared to 4.23 (SD = 8.7, median = 0, range 0–41) in intervention houses (Table 3; Fig. 2). In both unadjusted and adjusted models, the intervention did not affect culicine densities (unadjusted RR = 1.17, 95% CI: 0.83–1.63; adjusted RR = 1.12, 95% CI: 0.79–1.68) (Table 3 & Supplementary Table 3). Cooking location did not modify the effect of the intervention, and neither PM_2.5_ nor cooking location were associated with culicine densities (Supplementary Tables 4 & 6). Approximately 1% of *Cx. quinquefasciatus* mosquitoes collected in both control and intervention houses were blood-fed. Bloodmeal composition was similar between control and intervention houses (Supplementary Table 7).

### Synanthropic flies

We collected 1,022 synanthropic flies, of which 475 (46%) were Muscidae and 436 (43%) were Fanniidae. Mean synanthropic fly density was 2.77 (SD = 4.84, median = 1, range 0–28) in control houses compared to 0.91 (SD = 2.69, median = 0, range 0–10) in intervention houses (Table 3; Fig. 2, & Supplementary Table 2). In both unadjusted and adjusted analyses, the intervention was associated with a ≥ 65% reduction in synanthropic fly densities (unadjusted RR = 0.31, 95% CI: 0.22–0.45; adjusted RR = 0.35, 95% CI: 0.24–0.51) (Table3 & Supplementary Table 3).

**Table 3:** Effects of intervention on *Anopheles*, culicine, and synanthropic fly density.

Outcome	Control			Intervention			Unadjusted RR	P value	Adjusted RR	P value
	Visits	n	Mean (sd)	Visits	n	Mean (sd)				
*Anopheles*	271	143	0.5 (2.4)	296	213	0.7 (3.0)	0.92 (0.33, 2.55)	0.87	1.23 (0.51, 2.99)	0.65
*Culicines*	271	894	3.3 (5.4)	296	1251	4.2 (8.7)	1.17 (0.83, 1.63)	0.36	1.12 (0.79, 1.58)	0.53
*Synanthropic flies*	271	752	2.8 (4.8)	296	270	0.9 (2.7)	0.31 (0.22, 0.45)	< 0.001	0.35 (0.24, 0.51)	< 0.001

RR = rate ratio. Adjusted RRs for *Anopheles* and culicine mosquitoes were adjusted for maternal education, cracks or openings in house, bed net use, proximity to rice fields, and population density. Adjusted RRs for flies were adjusted for maternal education, cracks or openings in houses, and population density

Cooking indoors at follow-up was associated with a 62% reduction in fly densities (adjusted RR = 0.38, 95% CI: 0.27–0.55) (Supplementary Table 6), whereas densities were similar in intervention houses compared to control houses that cooked indoors (adjusted RR = 1.09, 95% CI: 0.53–2.25) (Supplementary Table 4). Indoor cooking at endline was also much higher among intervention households compared to control households (91% vs. 11.2%), suggesting that cooking location mediated much of the observed intervention effect on fly densities. Intervention kitchens were a mean of 2–3 m further away from latrines and rubbish pits, which are common fly breeding sites, compared to control kitchens (Table 2). PM_2.5_ in bedrooms was not associated with fly densities (Supplementary Table 6).

### Secondary outcomes: malaria and diarrheal disease

A total of 69 malaria cases (39 control, 30 intervention) were reported among mothers. Mean longitudinal prevalence of malaria was 8.1% (SD = 13.0) among mothers in the control group and 5.8% (SD = 12.1) among mothers in the intervention group. After controlling for potential confounders, the intervention was not associated with malaria prevalence among mothers (adjusted longitudinal prevalence ratio (LPR) = 0.92, 95% CI: 0.56–1.47) (Table 4). The results were similar when we restricted the analysis to only confirmed malaria cases. PM_2.5_ and cooking location were not associated with reported malaria risk in mothers (Supplementary Table 8).

**Table 4:** Effects of intervention on longitudinal prevalence of reported malaria in mothers and malaria and diarrhea in children.

Outcome	Control			Intervention			Adjusted Longitudinal Prevalence Ratio	*P* value
	obs. periods	*n* cases	mean longitudinal prevalence (sd)	obs. periods	*n* cases	mean longitudinal prevalence (sd)		
Malaria in mothers	471	39	8.1 (13.0)	512	30	5.8 (12.2)	0.92 (0.56, 1.47)	0.72
Malaria in mothers, confirmed	471	29	6.0 (12.0)	512	22	4.3 (9.9)	0.95 (0.53, 1.68)	0.86
Malaria in children	360	9	2.7 (9.8)	394	3	0.8 (4.7)	0.42 (0.09, 1.44)	0.20
Malaria in children, confirmed	360	6	1.8 (0.1)	394	3	0.8 (4.7)	0.56 (0.11, 2.36)	0.44
Diarrhea in children	359	32	8.8 (0.1)	397	30	7.4 (14.3)	0.96 (0.59, 1.56)	0.88

Obs. periods = periods of observation; Prevalence ratios adjusted for maternal education, number of people that slept in house, cracks or openings in house, mud floors, bed net use, elevation, proximity to rice fields, and population density

Participants reported 12 malaria cases in children (9 control, 3 intervention) during 754 periods of observation. Mean longitudinal prevalence of malaria was 2.7% (SD = 9.8) among children in the control group and 0.8% (SD = 4.6) among children in the intervention group. After controlling for potential confounders, malaria prevalence was lower children in the intervention arm compared to the control, although this effect estimate was imprecise and included the null (adjusted LPR = 0.42, 95% CI: 0.09–1.44) (Table 4). We did not evaluate the effects of PM_2.5_ on malaria prevalence in children because there were too few cases for which PATS + measurements were available (*n* = 5).

A total of 62 diarrhea episodes (32 control, 30 intervention) were reported among children during 756 follow-up periods of observation. Mean longitudinal prevalence of diarrhea was 8.8% (SD = 0.14) among children in the control group and 7.4% (SD = 14.27) among children in the intervention group. After controlling for potential confounders, the intervention was not associated with diarrhea prevalence in children (adjusted LPR = 0.96, 95% CI: 0.59–1.56) (Table 4). We observed a positive but not statistically significant association between each standard deviation increase in PM_2.5_ and diarrhea (adjusted LPR = 1.59, 95% CI: 0.86–2.82) (Supplementary Table 8).

## Discussion

This was the first randomized controlled trial to measure the impact of a clean cooking intervention on entomological parameters of vector-borne disease. We found no effect of the intervention on *Anopheles* and culicine density, bloodmeal composition, or *P. falciparum* infection in *Anopheles* mosquitoes. These findings indicate that the replacement of biomass fuels with LPG as part of the HAPIN household air pollution intervention likely did not affect exposure to these mosquitoes or the pathogens they transmit. Furthermore, synanthropic fly densities were substantially lower in intervention households, which may represent a co-benefit of the adoption of indoor cooking with cleaner-burning fuels in this setting. This study is important as cleaner cooking fuels are increasingly promoted and the effect of cooking fuels on vector behavior and exposure to vector-borne diseases remains poorly understood.

Additionally, we did not observe significant differences in reported malaria or diarrhea in mothers and children in the intervention arm compared to those in the control. While we hesitate to draw firm conclusions due to the low sample size and the fact that these were secondary outcomes, these findings were generally consistent with our primary entomological outcomes which showed similar mosquito densities and lower fly densities in intervention compared to control houses. A recent analysis of Demographic and Health Survey data for 85,263 children from 17 sub-Saharan African countries also found that cooking with cleaner fuels was not associated with increased malaria infection risk^26^.

*Anopheles* densities were higher in intervention houses compared to control houses that cooked indoors at follow-up, although this was an exploratory analysis and the difference was not statistically significant. This could potentially reflect increased repellant effects of PM_2.5_ or other pollutants from indoor biomass combustion, as we previously observed that indoor PM_2.5_ concentrations were associated with decreased household entry and host-seeking by lab-reared *Anopheles* mosquitoes in experimental conditions^8^. However, the low sample size of houses that cooked with biomass indoors in this study limited our ability to assess this potential effect. Additional studies could assess the effects of clean fuel adoption on *Anopheles* densities in other eco-epidemiological settings and where indoor biomass cooking is more common. Nevertheless, our results indicate that at least among households that primarily cook with biomass outdoors, switching to indoor cooking with cleaner fuels is unlikely to affect indoor *Anopheles* exposures.

A round of IRS was conducted approximately six months before the start of vector sampling for this study, which likely contributed to the low mosquito counts we observed. IRS coverage was not different by intervention status and should not have influenced vector densities across study arms. However, reduced vector densities may have affected study power and our ability to detect differences between control and intervention households. Self-reported secondary health outcomes were limited by the potential for recall and interviewer bias. We observed similar effects when we restricted the analysis to only confirmed malaria cases, but we were unable to confirm reported diarrhea cases. Further studies could include clinic-confirmed malaria and diarrhea cases to account for this possibility. Additionally, changes in vector exposure are considered the most likely mechanism through which cooking fuel changes could affect vector-borne disease^3^. However, we did not assess potential effects of this intervention on cockroaches, which can be vectors of enteric pathogens and might be influenced by changes in kitchen location^27^. Additionally, other effects of clean fuel adoption could influence infectious disease risk, such as improved immune function from reduced HAP exposure^28^. Follow-up studies could assess relationships between components of fuel combustion, immune and gut health biomarkers, and malaria and diarrhea risk.

These findings point to potential benefits of a more holistic approach to household environmental health. HAP interventions, water, sanitation and hygiene (WASH) interventions, and vector control interventions are all targeted at the household level but are traditionally delivered by siloed disease control programs^29^. Efforts to bridge these fields could capitalize on potential synergies between interventions (for example, fly control as a co-benefit of clean cooking interventions or mosquito control as a benefit of piped water interventions) in order to maximize household environmental health benefits.

## Electronic supplementary material

Below is the link to the electronic supplementary material.

Supplementary Material 1

## Data Availability

Deidentified study data and a data dictionary may be shared with interested researchers upon reasonable request to the corresponding author (IH).

## References

[1] Naghavi, M. et al. Global, regional, and National age-sex specific mortality for 264 causes of death, 1980–2016: a systematic analysis for the global burden of disease study 2016. *Lancet* **390**, 1151–1210 (2017). 28919116 10.1016/S0140-6736(17)32152-9 PMC5605883

[2] Hawkes, F. M., Dabiré, R. K., Sawadogo, S. P., Torr, S. J. & Gibson, G. Exploiting Anopheles responses to thermal, odour and visual stimuli to improve surveillance and control of malaria. *Sci. Rep.* **7**, 17283 (2017). 29229938 10.1038/s41598-017-17632-3 PMC5725576

[3] Biran, A., Smith, L., Lines, J., Ensink, J. & Cameron, M. Smoke and malaria: are interventions to reduce exposure to indoor air pollution likely to increase exposure to mosquitoes? *Trans. R. Soc. Trop. Med. Hyg.* **101**, 1065–1071 (2007). 17888474 10.1016/j.trstmh.2007.07.010

[4] Kweka, E. J. et al. Longitudinal evaluation of Ocimum and other plants effects on the feeding behavioral response of mosquitoes (Diptera: Culicidae) in the field in Tanzania. *Parasites Vectors*. **1**, 42 (2008). 18945343 10.1186/1756-3305-1-42 PMC2577633

[5] Bockarie, M. J., Service, M. W., Barnish, G., Momoh, W. & Salia, F. The effect of woodsmoke on the feeding and resting behaviour of Anopheles gambiae S.s. *Acta Trop.* **57**, 337–340 (1994). 7810390 10.1016/0001-706x(94)90080-9

[6] Abong’o, B., Gimnig, J. E., Omoke, D., Ochomo, E. & Walker, E. D. Screening eaves of houses reduces indoor mosquito density in rural, Western Kenya. *Malar. J.* **21**, 377 (2022). 36494664 10.1186/s12936-022-04397-y PMC9733111

[7] Vernède, R., van Meer, M. M. & Alpers, M. P. Smoke as a form of personal protection against mosquitos, a field study in Papua new Guinea. *Southeast Asian J. Trop. Med. Public Health*. **25**, 771–775 (1994). 7667730

[8] Hennessee, I. et al. Assessing the effects of cooking fuels on anopheles mosquito behavior: an experimental study in rural Rwanda. *The Am. J. Trop. Med. Hygiene. ***106****, **1196–1208 (2022). 10.4269/ajtmh.21-0997 PMC8991336 35189594

[9] Greenberg, B. *Flies and Disease. Vol. II. Biology and Disease.transmission* (Princeton University Press, 1973).

[10] Baana, K., Angwech, H. & Malinga, G. M. Ethnobotanical survey of plants used as repellents against housefly, Musca domestica L. (Diptera: Muscidae) in Budondo subcounty, Jinja district, Uganda. *J. Ethnobiol. Ethnomed.* **14**, 35 (2018). 29747673 10.1186/s13002-018-0235-6 PMC5946462

[11] Denloye, A. A., Teslim, K. O. & Fasasi, O. A. Insecticidal and repellency effects of smoke from plant pellets with or without D-allethrin 90 EC against three medical insects. *Journal Entomology* **1AD**; 3: 9–15 (2006).

[12] Torr, S. J., Mangwiro, T. N. & Hall, D. R. Shoo fly, don’t bother me! Efficacy of traditional methods of protecting cattle from Tsetse. *Med. Vet. Entomol.* **25**, 192–201 (2011). 21276027 10.1111/j.1365-2915.2010.00942.x

[13] Mortimer, K. et al. A cleaner burning biomass-fuelled cookstove intervention to prevent pneumonia in children under 5 years old in rural Malawi (the cooking and pneumonia Study): a cluster randomised controlled trial. *Lancet* **389**, 167–175 (2017). 27939058 10.1016/S0140-6736(16)32507-7 PMC5783287

[14] Madewell, Z. J. et al. Inverse association between dengue, Chikungunya, and Zika virus infection and indicators of household air pollution in Santa Rosa, Guatemala: A case-control study, 2011–2018. *PLOS ONE*. **15**, e0234399 (2020). 32559225 10.1371/journal.pone.0234399 PMC7304608

[15] Clasen, T. et al. Design and rationale of the HAPIN study: A multicountry randomized controlled trial to assess the effect of liquefied petroleum gas stove and continuous fuel distribution. *Environ. Health Perspect.* **128**, 47008 (2020). 32347766 10.1289/EHP6407 PMC7228119

[16] Quinn, A. K. et al. Fidelity and adherence to a liquefied petroleum gas stove and fuel intervention during gestation: the Multi-Country household air pollution intervention network (HAPIN) randomized controlled trial. *Int. J. Environ. Res. Public Health*. **18**, 12592 (2021). 34886324 10.3390/ijerph182312592 PMC8656791

[17] Johnson, M. et al. Exposure contrasts of pregnant women during the household air pollution intervention network randomized controlled trial. *Environ. Health Perspect.* **130**, 097005 (2022). 36112539 10.1289/EHP10295 PMC9480977

[18] Balakrishnan, K. et al. Exposure–response relationships for personal exposure to fine particulate matter (PM2·5), carbon monoxide, and black carbon and birthweight: an observational analysis of the multicountry household air pollution intervention network (HAPIN) trial. *Lancet Planet. Health*. **7**, e387–e396 (2023). 37164515 10.1016/S2542-5196(23)00052-9 PMC10186177

[19] ICF M&. Rwanda malaria indicator survey 2017. (2017). https://dhsprogram.com/pubs/pdf/MIS30/MIS30.pdf

[20] Hakizimana, E. et al. Spatio-temporal distribution of mosquitoes and risk of malaria infection in Rwanda. *Acta Trop.* **182**, 149–157 (2018). 29476726 10.1016/j.actatropica.2018.02.012

[21] Checkley, W. et al. Effects of cooking with liquefied petroleum gas or biomass on stunting in infants. *N Engl. J. Med.* **390**, 44–54 (2024). 38169489 10.1056/NEJMoa2302687 PMC12070489

[22] McCann, R. S. et al. Explaining variation in adult Anopheles indoor resting abundance: the relative effects of larval habitat proximity and insecticide-treated bed net use. *Malar. J.* **16**, 288 (2017). 28716087 10.1186/s12936-017-1938-1 PMC5514485

[23] Bell, M. et al. Comparing trap designs and methods for assessing density of synanthropic flies in Odisha, India. *Parasites Vectors*. **12**, 75 (2019). 30732628 10.1186/s13071-019-3324-z PMC6367737

[24] Zhou, G., Minakawa, N., Githeko, A. & Yan, G. Spatial distribution patterns of malaria vectors and sample size determination in spatially heterogeneous environments: A case study in the West Kenyan Highland. *J. Med. Entomol.* **41**, 1001–1009 (2004). 15605637 10.1603/0022-2585-41.6.1001

[25] Schmidt, W-P. et al. Sampling strategies to measure the prevalence of common recurrent infections in longitudinal studies. *Emerg. Themes Epidemiol.* **7**, 5–5 (2010). 20678239 10.1186/1742-7622-7-5 PMC2922204

[26] Woolley, K. E. et al. Cooking outdoors or with cleaner fuels does not increase malarial risk in children under 5 years: a cross-sectional study of 17 sub-Saharan African countries. *Malar. J.* **21**, 133 (2022). 35477567 10.1186/s12936-022-04152-3 PMC9044678

[27] Liu, J. et al. Intestinal pathogens detected in cockroach species within different food-related environment in Pudong, China. *Sci. Rep.* **14**, 1947 (2024). 38253647 10.1038/s41598-024-52306-x PMC10803747

[28] Lee, A., Kinney, P., Chillrud, S. & Jack, D. A systematic review of innate Immunomodulatory effects of household air pollution secondary to the burning of biomass fuels. *Ann. Glob Health*. **81**, 368–374 (2015). 26615071 10.1016/j.aogh.2015.08.006 PMC4758189

[29] Clasen, T. & Smith, K. R. Let the ‘A’ in WASH stand for air: integrating research and interventions to improve household air pollution (HAP) and water, sanitation and hygiene (WaSH) in low-income settings. *Environ. Health Perspect.* **127**, 025001 (2019). 30801220 10.1289/EHP4752 PMC6752941

