# Supplementary material for: Effects of indoor cooking with liquefied petroleum gas versus solid biomass on mosquito and fly density in households
Source: Sci Rep. 2025 Jun 4;15:19578. doi: 10.1038/s41598-025-03573-9 (PMC12137564; doi:10.1038/s41598-025-03573-9)
Supplement: Supplementary file 1 — Supplementary Material 1 [file 41598_2025_3573_MOESM1_ESM.docx]

**Supplementary Information:**

**Effects of indoor cooking with liquefied petroleum gas versus solid biomass on mosquito and fly density in households**

Ian Hennessee, PhD^1*^, Miles A. Kirby, PhD^2^, Xavier Misago, MS^3^, Jackie Mupfasoni, MS^3^, Jiantong Wang, MS^4^, Jean de Dieu Ntivuguruzwa,MS^5^, Florien Ndagijimana,BS^5^, Ghislaine Rosa,PhD^6^, Jennifer L. Peel, PhD^7^, Lance A. Waller, PhD,^1,4^ Joshua P. Rosenthal, PhD,^8ǂ^, Uriel Kitron^1,9^, Emmanuel Hakizimana, PhD^3^, & Thomas F. Clasen, PhD^1^

Ian Hennessee

Email: i.p.hennessee@gmail.com

Contents

[Supplementary Methods 2](#_Toc194569690)

[Intervention: 2](#_Toc194569691)

[Cooking practices: 2](#_Toc194569692)

[Housing characteristics: 2](#_Toc194569693)

[Vector control: 2](#_Toc194569694)

[Indoor conditions: 3](#_Toc194569695)

[Environmental characteristics: 3](#_Toc194569696)

[Follow-up time 3](#_Toc194569697)

[Supplementary tables 4](#_Toc194569698)

[Supplemental Table 1. 4](#_Toc194569699)

[Supplementary Table 2 4](#_Toc194569700)

[Supplementary Table 3. 6](#_Toc194569701)

[Supplementary Table 4 7](#_Toc194569702)

[Supplementary Table 5. 8](#_Toc194569703)

[Supplementary Table 6. 9](#_Toc194569704)

[Supplementary Table 7. 10](#_Toc194569705)

[Supplementary Table 8. 11](#_Toc194569706)

[References 11](#_Toc194569707)

# Supplementary Methods

Intervention: The primary exposure variable was intervention status. Intervention households had received LPG stoves and a consistent supply of LPG fuel as part of the HAPIN trial, whereas control households had not and were encouraged to continue cooking with traditional biomass fuels.^1^ Control households were considered the reference group. We also measured numerous other potential determinants of vector density. These included:

## Cooking practices:

- Fuel used in primary stove: at each sampling round, participants were asked whether they had cooked in the prior 24 hours and what type of fuel they had used for their primary stove (LPG, biomass, or other/unknown).
- Cooking location: participants were also asked where their primary cooking location was (outdoors or in a separate cooking structure, indoors in main house, or unknown).

## Housing characteristics:

- Number of people that slept in house night before: at each sampling round, participants were asked how many people slept in the house the night before.
- Presence of openings in windows, doors, and/or walls: study staff also visually observed the presence of any cracks or openings in windows, doors, or walls wider than 1cm, which could facilitate entry by mosquitoes or flies.
- Presence of open, water-holding containers: open, water-holding containers were counted and inspected for the presence of mosquito larvae and pupae.
- Toilet/ latrine covered: Staff observed whether houses had a toilet or latrine and if yes, whether it was covered.
- Distance from latrine to kitchen (m): study staff measured distance in meters from the latrine to the center of the primary cooking location.
- Distance from rubbish pile to kitchen (m): study staff asked participants where their primary rubbish or garbage disposal area was and measured distance in meters to the center of the primary cooking location.
- Domestic animals in compound: study staff visually observed the presence of any animals within the household compound.
- Feces in compound: study staff visually observed the presence of any animal or human feces in the household compound.
- Uncovered, cooked food in kitchen: study staff visually observed the presence of any cooked food that was either uncovered or not covered with a tight-fitting lid in the cooking area.

## Vector control:

- % of occupants that slept under net: at each sampling round, participants were asked how many individuals who slept in the house the night before slept under an insecticide treated bed net.
- Received IRS in last 12 months: Participants were asked whether their house had received IRS in the last 12 months prior to the survey, which was confirmed by observing government-issued IRS cards.
- Used insecticides or burned materials to repel mosquitoes or flies in last 24 hrs: participants were also asked if they had used insecticides or burned any materials to repel mosquitoes or flies in the 24 hours prior to the study team visit.

## Indoor conditions:

- PM_2.5_ concentrations in μg/m^3^: PM_2.5_ concentrations were measured in a subset of 144 participants’ bedrooms during each sampling round as described above. PM_2.5_ levels for each round were averaged as the mean concentrations from 4pm on the day of the initial visit until 10am on the day of the return visit the next morning.
- Temperature (°C): temperature was measured along with PM_2.5_ and averaged as described above.
- Percent relative humidity (RH): relative humidity was measured along with PM_2.5_ and temperature and averaged as described above.

## Environmental characteristics:

- Elevation (m): the elevation of each household was derived using a 30-meter digital elevation model (DEM) from the Shuttle Radar Topography Mission.^2^
- Distance (m) to closest rice fields and dams: a cloud-free 30-meter Landsat 8 L1 retrieval of the study area from March 04, 2020 was downloaded from the U.S. Geological Survey.^3,4^ We used supervised image classification to classify rice fields in the study area, following previously published methods.^5^ Briefly, we first classified potential wetlands where rice is typically grown using elevation and slope models.^6^ We then used principal components analysis (PCA) to remove correlation among bands 1 through 11.^7^ The first three components of the PCA analysis accounted for 99% of variation in the image. We used high resolution google earth imagery as well as in situ observations to manually assign training samples for six land-use classifications (rice, natural wetlands, dams/lakes, row-crop agriculture, scrubland, and settlements). We then employed supervised image classification in ArcMap version 10.8.1 (ESRI, Redlands, CA) and assessed model results via the kappa coefficient. We then calculated Euclidean distance from each study household to the nearest rice field using the *rgeos* package in R version 4.0.2.^8^ We used the same method to calculate distance from each house to the closest dam or lake.
- LST (°C): we downloaded 6km resolution monthly daytime land surface temperature (LST) averages from MODIS Terra MOD11B3 products in the NASA Earthdata portal.^9^ We calculated current and one-month lagged LST for each household at each study visit as the mean LST within a 2500m buffer area around each household using the *SP* and *Raster* packages in R.^10,11^
- Rainfall (mm): we downloaded monthly 6km resolution gridded rainfall estimates from The Climate Hazards group Infrared Precipitation with Stations (CHIRPS).^12^ We then estimated current and one-month lagged rainfall values for each household at each study visit as the mean rainfall within a 2500m buffer area around each household using the *SP* and *Raster* packages in R
- Population density/ km^2^: we downloaded high resolution (100m) gridded population density estimates from World Pop for the study area in 2019.^13^ We then estimated local population density for each study household as the mean population density within a 250m buffer area around each household using the *SP* and *Raster* packages in R.

Follow-up time

We calculated follow-up time for mothers as the number of days between the date of randomization into the trial to the last completed assessment. Follow-up time for children was calculated as the number of days from birth until the last completed assessment (e.g., visit B4). If one or more planned assessments were not completed, we subtracted the average follow-up time for that assessment from the overall follow-up time for each individual.

# Supplementary tables

| Supplemental Table 1. Proportion of *Anopheles* and culicine mosquitoes by collection method and location. | | | | | |
| --- | --- | --- | --- | --- | --- |
|  |  | **Total, n (%)** | **Bedrooms** | **Kitchen** | **Outdoors** |
| ***Anopheles*** | **CDC light trap** | 336 (94%) | 336 | – | – |
|  | **Prokopack** | 20 (6%) | 1 | 18 | 1 |
|  | **Sticky fly trap** | 0 (0%) |  |  |  |
|  |  |  |  |  |  |
| **Culicines** | **CDC light trap** | 1920 (90%) | 1920 | – | – |
|  | **Prokopack** | 164 (8%) | 71 | 80 | 13 |
|  | **Sticky fly trap** | 61 (3%) | – | 61 | – |

Supplementary Table 2: Vector density by intervention status

|  |  |  | Total | | Control | | Intervention | | P-value |
| --- | --- | --- | --- | --- | --- | --- | --- | --- | --- |
|  |  |  | n | Density (SD) | n | Density (SD) | n | Density (SD) |  |
| Mosquito density | |  |  |  |  |  |  |  |  |
|  | *Anopheles spp.* | | 356 | 0.63 (2.68) | 143 | 0.53 (2.35) | 213 | 0.72 (2.95) | 0.87 |
|  |  | *An. gambiae s.l.* | 291 | 0.51 (2.56) | 107 | 0.39 (2.21) | 184 | 0.62 (2.85) | 0.94 |
|  |  | *An. ziemanni* | 18 | 0.03 (0.24) | 8 | 0.03 (0.26) | 10 | 0.03 (0.23) | 0.86 |
|  | Culicines | | 2145 | 3.78 (7.32) | 894 | 3.30 (5.41) | 1251 | 4.23 (8.70) | 0.37 |
|  |  | *Culex quinquefasciatus* | 2048 | 3.61 (7.02) | 846 | 3.12 (5.10) | 1202 | 4.06 (8.39) | 0.41 |
|  |  | *Aedes spp.* | 11 | 0.02 (0.21) | 3 | 0.01 (0.10) | 8 | 0.03 (0.27) | 0.88 |
|  |  | *Mansonia spp.* | 11 | 0.02 (0.18) | 4 | 0.01 (0.12) | 7 | 0.02 (0.22) | 1.00 |
| Fly density | | |  |  |  |  |  |  |  |
|  | Synanthropic flies | | 1022 | 1.80 (3.98) | 752 | 2.77 (4.84) | 270 | 0.91 (2.69) | <0.001 |
|  |  | Muscidae | 475 | 0.84 (2.87) | 369 | 1.37 (3.22) | 106 | 0.36 (2.40) | <0.001 |
|  |  | Calliphoridae | 72 | 0.13 (1.23) | 62 | 0.23 (1.75) | 10 | 0.03 (0.29) | 0.22 |
|  |  | Fanniidae | 436 | 0.78 (2.19) | 297 | 1.10 (2.87) | 139 | 0.48 (1.21) | <0.001 |
|  |  | Sarcophagidae | 39 | 0.07 (0.65) | 24 | 0.09 (0.86) | 15 | 0.05 (0.38) | 0.99 |
|  | Domestic flies | | 8737 | 15.41 (27.73) | 4982 | 18.38 (35.27) | 3755 | 12.69 (17.90) | 0.03 |
|  |  | Drosophila | 6604 | 11.65 (25.25) | 3769 | 13.91 (32.13) | 2835 | 9.58 (16.41) | 0.03 |
|  |  | Psychodidae | 1031 | 1.82 (10.28) | 577 | 2.13 (13.91) | 454 | 1.53 (5.06) | 0.99 |

| Supplementary Table 3. Unadjusted and adjusted effects of intervention and other variables on *Anopheles,* culicine, and synanthropic fly densities | | | | | | | | | | | | |
| --- | --- | --- | --- | --- | --- | --- | --- | --- | --- | --- | --- | --- |
|  | ***Anopheles* density** | | | | **Culicine density** | | | | **Synanthropic fly density** | | | |
|  | *Unadjusted* | | *Adjusted* | | *Unadjusted* | | *Adjusted* | | *Unadjusted* | | *Adjusted* | |
|  | *RR (95% CI)* | *P value* | *RR (95% CI)* | *P value* | *RR (95% CI)* | *P value* | *RR (95% CI)* | *P value* | *RR (95% CI)* | *P value* | *RR (95% CI)* | *P value* |
| Intervention *(ref = control)* | 0.92 (0.33, 2.55) | 0.87 | 1.23 (0.51, 2.99) | 0.65 | 1.17 (0.83, 1.63) | 0.37 | 1.12 (0.79, 1.58) | 0.53 | 0.31 (0.22, 0.45) | <0.001 | 0.35 (0.24, 0.51) | <0.001 |
| Maternal education *(ref = primary or less)* | 0.24 (0.09, 0.66) | 0.01 | 0.51 (0.19, 1.4) | 0.19 | 0.7 (0.49, 0.98) | 0.04 | 0.64 (0.45, 0.92) | 0.02 | 0.49 (0.33, 0.74) | <0.001 | 0.64 (0.43, 0.96) | 0.03 |
| Openings in house *(ref = no)* | 7.96 (2.4, 26.39) | <0.001 | 1.86 (0.5, 6.96) | 0.36 | 0.83 (0.57, 1.19) | 0.31 | 0.9 (0.57, 1.41) | 0.63 | 1.72 (1.11, 2.67) | 0.01 | 1.25 (0.75, 2.08) | 0.38 |
| % of occupants slept under net | 2.39 (0.87, 6.57) | 0.09 | 4.02 (1.48, 10.9) | 0.01 | 1.27 (0.86, 1.88) | 0.23 | 1.22 (0.81, 1.85) | 0.35 | – | – | – | – |
| Rice field within 2km *(ref = no)* | 20.38 (7.53, 55.12) | <0.001 | 4.27 (0.77, 23.55) | 0.10 | 2.16 (1.44, 3.23) | <0.001 | 1.76 (0.77, 4.02) | 0.18 | – | – | – | – |
| Population density / sq. km^ǂ^ | 0.05 (0.01, 0.2) | <0.001 | 0.07 (0.02, 0.29) | <0.001 | 1.09 (0.91, 1.29) | 0.35 | 1.11 (0.89, 1.39) | 0.36 | 0.7 (0.56, 0.86) | <0.001 | 0.91 (0.7, 1.18) | 0.49 |
| RR= Rate Ratio; ǂ = continuous variables scaled to standard deviations; | | | | | | | | | | | | |

| Supplementary Table 4. Effects of the intervention on vector density by cooking location | | | | | | | | | |
| --- | --- | --- | --- | --- | --- | --- | --- | --- | --- |
|  |  | Control | | Intervention | | Effect estimates | | | |
|  | Primary cooking location* | n obs | Mean (SD) | n obs | Mean (SD) | Unadjusted RR | P value | Adjusted RR | P value |
| *Anopheles* | Inside main house | 56 | 0.16 (0.83) | 309 | 0.6 (2.70) | 1.86 (0.18, 19.28) | 0.60 | 3.66 (0.92, 14.59) | 0.07 |
|  | Outdoors/ separate | 262 | 0.72 (2.99) | 34 | 0.97 (3.25) | 0.73 (0.10, 5.47) | 0.76 | 1.09 (0.31, 3.83) | 0.89 |
| Culicines | Inside main house | 56 | 4.2 (6.38) | 309 | 4.71 (10.46) | 0.86 (0.49, 1.49) | 0.59 | 0.75 (0.43, 1.3) | 0.31 |
|  | Outdoors/ separate | 262 | 3.16 (5.32) | 34 | 3.82 (5.73) | 1.11 (0.56, 2.2) | 0.76 | 1.08 (0.57, 2.07) | 0.81 |
| Synanthropic flies | Inside main house | 56 | 1.11 (2.56) | 309 | 0.82 (2.44) | 0.72 (0.37, 1.43) | 0.35 | 1.09 (0.53, 2.25) | 0.81 |
|  | Outdoors/ separate | 262 | 2.79 (4.86) | 34 | 2 (6.82) | 0.47 (0.2, 1.06) | 0.07 | 0.64 (0.29, 1.41) | 0.27 |
| * location of primary cooking stove used in 24 hrs before vector sampling visits; n obs = number of observations; Adjusted Rate ratios (RR) for *Anopheles* and culicines adjusted for maternal education, number of people that slept in house, cracks or openings in house, mud floors, bed net use, elevation, proximity to rice fields, and population density. Adjusted RRs for synanthropic flies adjusted for same variables except number of people that slept in house and rice field proximity | | | | | | | | | |

| Supplementary Table 5. PM_2.5_ and *Anopheles* density by cooking location | | | | |
| --- | --- | --- | --- | --- |
|  | **Control houses** | | | |
|  |  | PM2.5 (μg/m^3^) | | *Anopheles* density |
|  | n obs | Mean (SD) | Median (IQR) | Mean (SD) |
| Inside main house | 17 | 61.17 (120.2) | 27.18 (17.96, 48.02) | 0 (0) |
| Outdoors/ separate | 74 | 36.94 (61.32) | 20.92 (14.26, 29.28) | 0.45 (1.64) |
|  | **Intervention houses** | | | |
|  |  | PM2.5 (μg/m^3^) | | *Anopheles* density |
|  | n obs | Mean (SD) | Median (IQR) | Mean (SD) |
| Inside main house | 120 | 24.43 (28.63) | 17.41 (11.81, 25.52) | 0.16 (0.88) |
| Outdoors/ separate | 7 | 15.75 (8.27) | 13.94 (10.45, 15.71) | 2.14 (5.67) |

| Supplementary Table 6. Effects of PM_2.5_ and cooking location on vector density | | | | | | | |
| --- | --- | --- | --- | --- | --- | --- | --- |
|  |  | *Anopheles* mosquitoes | | Culicine mosquitoes | | Synanthropic flies | |
|  |  | *RR (95% CI)* | *P value* | *RR (95% CI)* | *P value* | *RR (95% CI)* | *P value* |
| PM2.5 (μg/m^3)^ | | 0.65 (0.15, 2.82) | 0.57 | 0.98 (0.77, 1.26) | 0.90 | 0.96 (0.72, 1.28) | 0.78 |
| Cooking location at follow-up | |  |  |  |  |  |  |
|  | *Indoor* | –* | – | 1.25 (0.92, 1.69) | 0.15 | 0.38 (0.27, 0.55) | <0.001 |
|  | *Outdoor/separate (ref)* | – | – | – | – | – | – |
| *The effect of cooking location on *Anopheles* density was not evaluated due to low sample sizes. Rate ratios (RR) for *Anopheles* and culicine mosquitoes adjusted for maternal education, number of people that slept in house, cracks or openings in house, mud floors, bed net use, elevation, proximity to rice fields, and population density. RRs for flies adjusted for same variables except number of people that slept in house and rice field proximity | | | | | | | |

| Supplementary Table 7. Bloodfeeding and sporozoite infections among *Anopheles* and *Culex* mosquitoes, by intervention status | | | | | |
| --- | --- | --- | --- | --- | --- |
|  |  |  | Control | Intervention | P-value |
|  |  |  | n (%) | n (%) |  |
| *Anopheles* | |  |  |  |  |
|  | Bloodfed |  | 33/204 (16.2) | 11/213 (5.2) | 0.344 |
|  |  | Human | 22/33 (66.7) | 6/11 (54.5) | 0.631 |
|  |  | Bovine | 2/33 (6.1) | 0/11 (0.0) |  |
|  |  | Mixed | 7/33 (21.2) | 4/11 (36.4) |  |
|  |  | Other | 2/33 (6.1) | 1/11 (9.1) |  |
|  | Pf + |  | 1/33 (0.5) | 0/11 (0.0) | 0.983 |
| *Culex* |  |  |  |  |  |
|  | Bloodfed |  | 9/1071 (0.8) | 20/1602 (1.2%) | 0.321 |
|  |  | Human | 1/9 (11.1) | 1/20 (5.0) | 0.459 |
|  |  | Bovine | 0/9 (0.0) | 1/20 (5.0) |  |
|  |  | Mixed | 7/9 (77.8) | 11/20 (55.0) |  |
|  |  | Other | 1/9 (11.1) | 7/20 (35.0) |  |

| Supplementary Table 8. Adjusted effects of PM_2.5_ and cooking location on reported health outcomes | | | | | | | |
| --- | --- | --- | --- | --- | --- | --- | --- |
|  |  | Malaria in mothers | | Malaria in children | | Diarrhea in Children | |
|  |  | *LPR (95% CI)* | *P value* | *LPR (95% CI)* | *P value* | *LPR (95% CI)* | *P value* |
| PM_2.5_ (μg/m^3)^ | | 0.95 (0.45, 1.32) | 0.83 | NA* |  | 1.59 (0.86, 2.82) | 0.12 |
| Cooking location at follow-up | |  |  |  |  |  |  |
|  | *Indoor* | 1.11 (0.7, 1.74) | 0.66 | 0.38 (0.08, 1.28) | 0.15 | 0.95 (0.59, 1.51) | 0.81 |
|  | *Outdoor/separate (ref)* |  |  |  |  |  |  |
| *Effect of PM_2.5_ on malaria in children not evaluated because too few cases were observed among children with PM2.5 measurements (n=5). Longitudinal prevalence ratios (LPR) for malaria adjusted for maternal education, number of people that slept in house, cracks or openings in house, mud floors, bed net use, elevation, proximity to rice fields, and population density. LPRs for diarrhea adjusted for same variables except number of people that slept in house and rice field proximity. | | | | | | | |

# References

1. Clasen T, Checkley W, Peel JL, et al. Design and Rationale of the HAPIN Study: A Multicountry Randomized Controlled Trial to Assess the Effect of Liquefied Petroleum Gas Stove and Continuous Fuel Distribution. *Environ Health Perspect* 2020; **128**(4): 47008.

2. RCMRD. Rwanda SRTM 30 meters [online]. 2015. <http://geoportal.rcmrd.org/layers/servir%3Arwanda_srtm30meters> (accessed April 30 2020).

3. USGS. Earth Explorer. 2021. <https://earthexplorer.usgs.gov/> (accessed April 28 2021).

4. Roy DP, Wulder MA, Loveland TR, et al. Landsat-8: Science and product vision for terrestrial global change research. *Remote sensing of Environment* 2014; **145**: 154-72.

5. Diuk-Wasser MA, Touré MB, Dolo G, et al. Effect of rice cultivation patterns on malaria vector abundance in rice-growing villages in Mali. *The American journal of tropical medicine and hygiene* 2007; **76**(5): 869-74.

6. Mahdavi S, Salehi B, Granger J, Amani M, Brisco B, Huang W. Remote sensing for wetland classification: a comprehensive review. *GIScience & Remote Sensing* 2018; **55**(5): 623-58.

7. Lei TC, Wan S, Chou TY. The comparison of PCA and discrete rough set for feature extraction of remote sensing image classification – A case study on rice classification, Taiwan. *Computational Geosciences* 2008; **12**(1): 1-14.

8. Bivand R, Rundel C, Pebesma E. rgeos: interface to geometry engine-open source (GEOS). *R package version 03-26* 2017.

9. NASA. NASA Earthdata. 2021. <https://search.earthdata.nasa.gov/search> (accessed October 20 2020).

10. Hijmans RJ, Van Etten J, Cheng J, et al. Package ‘raster’. *R package* 2015; **734**.

11. Pebesma E, Bivand RS. S classes and methods for spatial data: the sp package. *R news* 2005; **5**(2): 9-13.

12. Funk C, Peterson P, Landsfeld M, et al. The climate hazards infrared precipitation with stations—a new environmental record for monitoring extremes. *Scientific Data* 2015; **2**(1): 150066.

13. Lloyd CT, Chamberlain H, Kerr D, et al. Global spatio-temporally harmonised datasets for producing high-resolution gridded population distribution datasets. *Big Earth Data* 2019; **3**(2): 108-39.
